# A Low-Latency Optimization of a Rust-Based Secure Operating System for Embedded Devices

**DOI:** 10.3390/s22228700

**Published:** 2022-11-10

**Authors:** Ioana Culic, Alexandru Vochescu, Alexandru Radovici

**Affiliations:** Computer Science Department, Automatic Control and Computer Science Faculty, University Politehnica of Bucharest, 060042 Bucharest, Romania

**Keywords:** embedded, security, real-time, low-latency, eBPF, rust, RTOS, Tock

## Abstract

Critical systems such as drone control or power grid control applications rely on embedded devices capable of a real-time response. While much research and advancements have been made to implement low-latency and real-time characteristics, the security aspect has been left aside. All current real-time operating systems available for industrial embedded devices are implemented in the C programming language, which makes them prone to memory safety issues. As a response to this, Tock, an innovative secure operating system for embedded devices written completely in Rust, has recently appeared. The only downside of Tock is that it lacks the low-latency real-time component. Therefore, the purpose of this research is to leverage the extended Berkeley Packet Filter technology used for efficient network traffic processing and to add the low-latency capability to Tock. The result is a secure low-latency operating system for embedded devices and microcontrollers capable of handling interrupts at latencies as low as 60 µs.

## 1. Introduction

The main characteristic of a real-time system is that it needs to ensure a deterministic response to a trigger in a specific time interval. Plenty of fields, such as manufacturing, transport, or healthcare, rely on machines that can ensure a real-time response in critical operations [1]. For example, devices such as autonomous robots or drones rely on real-time processing as their movements depend on the inertial data coming from the incorporated sensors. In the case of a drone, the embedded microcontroller reads data from accelerometers and gyroscopes to determine the system’s future position and adapt the rotors’ position and speed to ensure that the device maintains a correct path [2].

The highest efficiency in handling real-time operations is achieved by running bare-metal software on top of the hardware device [3]. This approach easily ensures a time-deterministic response to any trigger [4] and that any interrupt can be handled without any overhead instantly. However, with the advancements related to operating systems and low-level programming approaches, deploying bare-metal applications nowadays seems to be an obsolete approach with multiple downsides. A large percentage of the systems deployed in both industrial and consumer environments run an operating system, on top of which, applications are deployed, rather than running one single piece of bare-metal software.

Deploying applications on top of an operating system, even for embedded devices, has the following advantages:

- Fast development—building standalone applications is faster and easier than bundling them with drivers and other system components. This approach allows the developer to focus on developing a working application, rather than on the communication between the app and the rest of the necessary components.
- Easy updates—updating a component of a bare-metal system involves rebuilding all of the components to obtain the final binary and reflashing the complete system. In addition, many components are tightly coupled and one change can lead to numerous necessary adaptations. This makes the development process longer and brings a large amount of overhead. Operating systems, on the other hand, are modular, and changes are made faster.
- Multiple applications—as gadgets and embedded systems become more complex, they handle multiple tasks, which can be divided into different applications (e.g., a smartwatch that monitors users’ sleep patterns and also displays phone notifications). An operating system is capable of handling multiple applications (processes) and, if possible, ensuring memory separation between different processes.

On the other hand, the major downside of running an operating system rather than bare-metal software is the overhead. The modular implementation of operating systems introduces overheads related to handling specific system calls, the existence of process schedulers, etc. For instance, interrupt handling in the case of an operating system involves context switches from the user space to kernel space, which increases the response time to external triggers. However, there are some specific operating systems that are implemented in a different manner so that they can support real-time operations and be integrated in critical installations.

Real-time embedded operating systems (RTOSs) are designed for low-capability microcontrollers and enable time deterministic responses to specific triggers for embedded systems while offering more modularity than a bare-metal approach [5]. While the best low latency is obtained when running bare-metal interrupt handlers, RTOSs are also able of obtaining a low latency. Generally, real-time operating systems rely on kernel pre-emption to ensure that interrupts are handled as soon as they are fired, but a context switch overhead is still present. In addition, if the kernel pre-emption mechanism is not correctly implemented, it can lead to kernel lock and process starvation. Therefore, an important aspect when writing drivers and applications to run on top of an RTOS is to ensure that all tasks are deterministic [6].

In addition, all RTOSs are written in C, as they need low-hardware control. However, running C code has a high potential for security issues. The industry states that around 70% of vulnerabilities are due to memory safety issues [7,8]. This is mainly because of how C implements memory operations that can easily lead to buffer overflows or control-flow attacks [9]. In addition, initially, real-time systems were designed to run on isolated devices, which are devices that are not connected to a network. At most, these devices would implement simple communication protocols only for exchanging data. When running on a connected device, such systems lack the necessary protection mechanisms and are prone to security attacks [10].

The need for fast responses can also be found at the network stack level, where packets need to be sent, processed, and, most importantly, filtered, in real-time. In Linux systems, the need of filtering packets is handled by implementing the extended Berkeley Packet Filter (eBPF) technology. eBPF is a kernel sandbox that runs custom code [11] and that was first used to apply custom filters to network packets in order to increase the speed for network applications. This approach is suitable for Linux as it enables custom code to be run at the kernel layer without the need to include it as an individual driver, which would offer unrestricted access to the system. Therefore, this process is more efficient and also more portable.

With the advent of the Internet of Things, real-time systems have become integrated into an increasing number of various connected devices covering critical fields such as health, agriculture, and transportation [12]. Therefore, the security of these devices is extremely important [13] and attacks such as Mirai have proved that multitude of devices can be affected [14].

To this end, this paper contributes to securing real-time systems by proposing a low-latency add-on to an RTOS written in Rust. Therefore, our purpose is to improve the real-time components of an operating system written in another language than C. For this, we used a novel operating system for embedded devices, which was written in Rust to achieve a high level of safety, to which, we added a bpf-based real-time component. The result is a secure operating system that runs a non-pre-emptive kernel and can handle real-time interrupts.

This paper is structured into six sections as follows: Section 2 describes the characteristics of some of the most popular operating systems for embedded devices; Section 3 gives an overview of the approach that we suggest for obtaining a real-time secure embedded operating system; Section 4 details the implementation of the proposed approach; Section 5 presents the results of the tests ran for validating the solution; Section 6 draws the conclusions based on the obtained results and presents the future steps to be taken into account for this research.

## 2. Operating Systems for Embedded Devices

Embedded devices have various characteristics and constraints based on the products that they are integrated with and their usage. For example, in the case of a smart tracker, we emphasize the need for a small-sized MCU and low power consumption. On the other hand, in the case of an industrial installation, the previous characteristics are not that important when compared with other requirements, such as robustness and real-time responses. Other requirements to be taken into account are network and Internet connection and transfer speed, accurate sensor readings, scalability, mobility, and security [15].

Based on the aforementioned constraints, two different processing units can be used: single-board computers (SBCs) and microcontroller boards (MCBs) [16]. SBCs are small-sized computers capable of executing multiple tasks and running a full-fledged operating system that has capabilities such as virtual memory or a complex process scheduler. They can run Linux or Windows distributions and execute multiple parallel processes. On the other hand, MCBs have reduced computing capabilities and can run only one piece of software, usually called firmware [17]. Due to this, MCBs are better suited in real-time systems.

Newer microcontrollers have started to become more and more powerful, being able to run more complex firmware. This work refers to such systems.

Whereas SBCs use regular operating systems that have certain adjustments for embedded systems, in the case of newer MCBs, specific operating systems are required. These OSs leverage the devices’ capability of running real-time tasks and, thus, are called real-time operating systems (RTOSs).

### 2.1. The Characteristics of Real-Time Operating Systems

Real-time operating systems are designed to efficiently manage resources for microcontroller devices with reduced capabilities and to handle multiple concurrent tasks. In addition, the scheduling mechanisms employed are adapted to support real-time operations. This is usually implemented based on kernel pre-emption and fast context switching [18,19].

In this context, we can enumerate the following main characteristics of RTOSs, based on which, evaluation metrics can be identified:

- Responsiveness—this is one of the main characteristics of real-time operating systems as they need to ensure the handling of multiple events in a clearly defined time frame. In addition, the response time should be easy to predict. The lower the interrupt handling time, the higher the system’s responsiveness is considered to be.
- Determinism—system determinism is presented in close relation to its responsiveness. Determinism implies that each task is executed at a specific time that can be computed for a clearly defined period. Together with responsiveness, they give an operating system the real-time characteristic [6].
- Multitasking—while MCBs can run only one task at once, the operating system has the ability to handle multiple operations and switch between what seems to appear as different processes. The OS needs the ability to efficiently switch between and pre-empt different tasks to ensure that critical operations are handled in real-time [20].
- Safety—with multiple individual tasks running on the system, they need to communicate and access hardware resources. The operating system needs to ensure that tasks do not overlap in accessing resources and that no non-deterministic behaviour can happen [21].

All of the characteristics presented above can be reduced to two main important aspects, which are response time and safety. For RTOSs, the first one has been a vital characteristic, as reviews by F. Javed et al. [22] and I. Ungureanu [5] outline, while the safety of RTOSs is being researched more and more at the moment.

### 2.2. Production Grade RTOSs

A market study carried out by AspenCore outlines the industry trends regarding embedded operating systems usage [23]. The latest report published in 2019 identifies FreeRTOS [24] as the most used real-time operating system. Other RTOSs enumerated in the report are VxWorks [25] and Keil (RTX) [26]. Other operating systems used in commercial applications and by the hobbyist community are the Zephyr Project [27,28] and RIOT [29]. Although they are not present in the classification presented by AspenCore, both operating systems have a large community of contributors. For instance, RIOT is used by Continental in one of their modules for vehicle access [30].

#### 2.2.1. FreeRTOS

FreeRTOS is an open source (GPL license) operating system for microcontrollers that was developed in 2002. It is fully developed in C and only supports C applications. The applications are compiled as static libraries included in the kernel. Therefore, including or removing an app from the system requires recompiling the full system image, including the kernel. FreeRTOS’s implementation relies on a dynamic pre-emptive priority-based scheduler and uses mutexes and semaphores for the tasks’ synchronization.

Starting with 2017, FreeRTOS support is offered by Amazon and, since then, it is often referred to as Amazon FreeRTOS [31].

#### 2.2.2. VxWorks

VxWorks is a real-time operating system developed by Wind River. In contrast to FreeRTOS, VxWorks is not fully open source, and only has some public components. Similar to FreeRTOS, it is written in C, but it supports applications written in various languages such as Python and Rust [25,32]. It is built as a modular system where additional libraries can be integrated to provide functions such as file sharing or networking. The kernel implements a pre-emptive round-robin algorithm for scheduling tasks, where each task has a priority between 0 and 255 [33].

The main advantage of VxWorks is that it supports container-based application deployment.

#### 2.2.3. RIOT

RIOT is another open source (LGPL license) real-time operating system for constrained embedded devices [34]. In a comparison made by S. Challouf et al. [35], RIOT is more efficient from both a memory and power consumption point of view when compared to FreeRTOS. To reduce its footprint and processing, it has a micro-kernel architecture where only the main features are implemented in the core kernel, while additional capabilities can be integrated as external libraries. This is similar to VxWorks’ approach. In addition, similar to the previous two operating systems presented above, RIOT is entirely written in C and supports applications written in C/C++. It also relies on a pre-emptive priority-based scheduler to provide real-time capabilities [22].

#### 2.2.4. The Zephyr Project

Zephyr is an open source RTOS developed as a Linux Foundation Project. It is designed as an operating system for constrained devices and supports various architectures, from ARM to Intel and RISC-V. It is a configurable OS; thus, developers can choose from multiple scheduling algorithms or kernel services based on their needs. Among the supported services are multi-threading, interrupt handlers, inter-thread synchronization, and power management. In addition, Zephyr implements memory protection for devices supporting MMU. The Zephyr Project is very popular, being supported by companies such as Intel, Google, and NXP.

### 2.3. Operating Systems Not Written in C

The three operating systems presented in the section above have several common features, which are also common to most of the existing real-time operating systems. Out of all, one common characteristic that stands out is that all RTOSs are written in C. Furthermore, even when referring to general-purpose operating systems, all of them are also written in C. This is mainly because there is no other mainstream programming language that gives developers the control that they need over the system. To be suitable for writing an operating system, a language has to compile to native code and run without a runtime and a garbage collector. The downside of this is that C programs, whether operating systems or applications, are prone to security issues due to the risk of bad memory operations [9,36].

The only programming language that has an implementation that gives the necessary control over the system is Rust. Developed by Mozilla Research and released in 2015, Rust is an open source programming language that focuses on security and efficiency [37]. What makes it suitable for systems programming is the lack of a runtime and garbage collector. Furthermore, it is proven to be type-safe and memory-safe [38]. What makes Rust different from system languages is that it provides almost all of the high-level language features, such as automatic memory management and reference management, without the need for a garbage collection or a runtime library.

Considering Rust’s characteristics, we can identify operating systems written exclusively in this programming language. While most of them started as a research topic and are not completely implemented, three stand out as being stable and ready to install by other users: Redox [39], Tock [40], and Hubris [41]. Due to the novelty related to Rust and the constant changes that it undergoes, these three operating systems are still under development as new features are added to them.

While the three OSs mentioned above are fully functional and used in the industry, we can identify multiple other operating systems and RTOSs fully written in Rust (e.g., Drone OS [42], Bern RTOS [43], Theseus [44]), most of them being research or hobby projects. This is why we choose to focus on these three as we consider them to be the most mature ones.

#### 2.3.1. Redox

Redox is a general-purpose operating system written in Rust. It is an open source Unix-like operating system written in Rust. It behaves like any general-purpose operating system and supports Unix commands. It relies on relibc, which is the libc library written in Rust. This is how Redox maintains the appearance of any Unix-like OS [39]. Redox does not follow the POSIX standard. For instance, resources are not represented as files, as in all POSIX systems, but as URLs [45].

#### 2.3.2. Hubris

Designed for deeply embedded chips, Hubris is more of a scheduler and IPC framework [46] than an actual operating system. Its main focus is to run on chips that users never interact with, such as root-of-trust devices, encryption devices, or signal processors. Hubris provides memory protection out of the box, requiring chips to provide hardware memory protection mechanisms. At the time of writing, it runs on ARM Cortex-M with MPU systems.

The kernel is composed of a scheduler and an IPC mechanism. All drivers are normal user space applications that are allowed to directly access peripherals.

#### 2.3.3. Tock

Tock is an operating system for ARM Cortex-M and RISC-V-based microcontrollers that is designed to safely run multiple concurrent applications [40,47]. Tock is an open source OS that is entirely developed in Rust to guarantee a high level of safety. Furthermore, it implements out-of-the-box memory isolation mechanisms and software fault isolation, which make it a good candidate for running secure sensitive applications [48,49].

Tock is specially designed for embedded devices, which makes it less prone to security attacks related to memory management. However, in the context that we present, the main downside of Tock is that, due to its architecture, it does not support real-time operations. The Tock kernel is not a pre-emptive one, meaning that driver actions cannot be interrupted.

## 3. The Approach

The purpose of the work presented in this paper is to propose a real-time component for an embedded operating system that can also ensure a high degree of security. After a short review of the existing technologies dedicated to embedded applications, we identified that all classic RTOSs have a major security penalty that is tightly related to the fact that all of these operating systems are written in C. Due to the way in which memory management is implemented in C, buffer overflows and other similar attacks are frequent.

On the other hand, Hubris and Tock, embedded operating systems that are entirely written in Rust, are not affected by most of the C memory management-related security threats. The only aspect that they are lacking is real-time support. Therefore, we aimed to enhance Tock and integrate a real-time mechanism to achieve a high level of security while ensuring the fast handling of specific events. We decided to perform our research using Tock, as Hubris is more of a task scheduler than an actual operating system. Due to it allowing direct hardware access for applications, Hubris presents several security risks, such as memory leaks between application restarts or the possibility of an application resetting the system.

### 3.1. Security Enforcement in Tock

The safety of the Tock operating system relies on three main implementation characteristics:

1. The kernel is written in Rust, with the number of unsafe code lines reduced to the minimum.
2. Drivers are divided into two layers: low-level drivers that have direct access to hardware and capsules, and upper-level drivers that abstract the low-lever ones and are not allowed to use unsafe Rust. Most of the development is carried out at the capsule layer.
3. The usage of hardware memory protection to restrict the applications from accessing memory outside their address space.

Rust is a programming language initially developed by Mozilla Research with the purpose of achieving a high level of security. Furthermore, in time, Rust evolved and also became a highly efficient language built with safety from the ground up. The current version of Rust relies on a powerful compiler that has the capability to manage memory at the compile time without requiring a garbage collector, which other modern programming languages rely on [40]. This makes Rust suitable for secure systems programming.

Rust is a type-safe language that also prevents programmers from introducing bugs related to memory management or data races. Potential issues, such as null pointer access or unsynchronized access to shared memory, are identified at compile time. To enforce this level of safety, Rust relies on an ownership and borrowing mechanism where every variable has a clearly defined lifetime and either only one mutable reference to it, or only read-only references to it [50].

Leveraging all Rust’s characteristics presented above, Tock defines itself as an operating system for microcontrollers built with security in mind.

In contrast to other operating systems for low-power embedded platforms, Tock clearly isolates kernel memory from application memory. It relies on the memory protection unit (MPU) that most advanced microcontrollers have implemented. Furthermore, the kernel, written in Rust, can run applications written in any programming language, and malicious ones have no negative impact on the system’s integrity [49,51].

**Figure 1:**
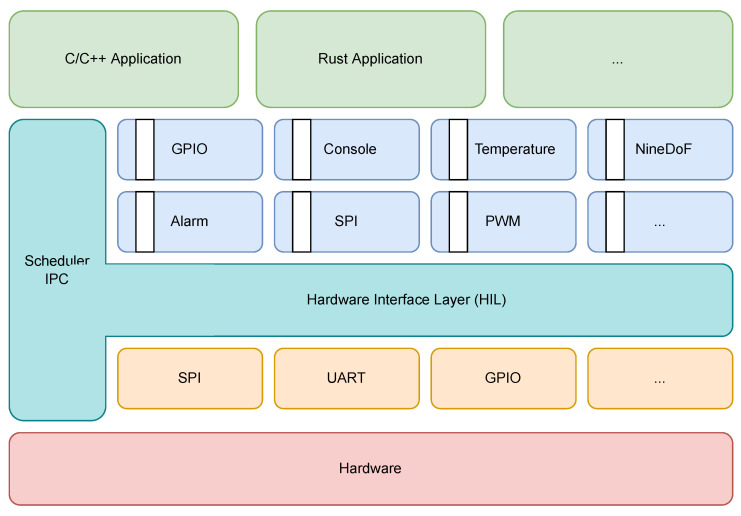
The Tock stack.

Figure 1 illustrates the Tock implementation stack outlining the separation between the kernel space and user space [52]. Furthermore, the kernel is divided into three main components, so the system is modular:

- Peripheral drivers—they are similar to the drivers in general-purpose operating systems and are required to control the peripherals. They expose a hardware interface layer (HIL) to the upper layers.
- Core kernel—this is the kernel per se, implementing the memory management, process scheduler, and inter-process communication mechanisms.
- Capsules—these are upper-level drivers, acting as an intermediate layer between the peripheral drivers and the user space.

According to the Tock’s design, the implementation of the peripheral drivers and the core kernel can contain unsafe Rust code. Unsafe blocks of code are necessary for certain operations that cannot be implemented according to the rules enforced by the Rust compiler. All blocks marked as unsafe are ignored by the compiler’s checks and, if not carefully written, can introduce security vulnerabilities. This is why the Tock team struggles to keep unsafe blocks to a minimum. However, the kernel sometimes needs to carry out some sensitive operations, such as writing I/O registers [49].

Applications run in the user space and are compiled completely separately from the kernel. The advantage of this approach is that deploying the kernel and applications can be carried out separately and applications are installed on top of Tock in a similar manner to general-purpose systems. In addition, applications are running in an unprivileged mode, having no direct access to the hardware. Tock uses system calls and upcalls to enable user space communication with the kernel. System calls are used by processes to ask for services from the kernel and upcalls are used to send data from the kernel to the user space. The Tock kernel is asynchronous and relies on asynchronous behaviour to handle multiple system calls in parallel. While this clear separation between the kernel and user space has safety advantages, it introduces delays in the process of interrupt handling, as applications cannot directly expose interrupt handlers.

Another Tock characteristic is that the kernel is non-pre-emptive. Due to this architecture, interrupts are handled only as bottom halves. Allowing top-half interrupt handlers would mean that drivers could be interrupted. Tock capsules rely on the fact that they are executing in a single thread and that nothing executes in parallel with them. This approach avoids using synchronization mechanisms and provides a deterministic behavior within the driver. This is why the current Tock implementation cannot support hard or soft real-time operations.

### 3.2. Possible Approaches

Considering the Tock stack, one of the overheads that we identified as preventing the operating system from running low-latency real-time operations is the context switch from kernel to application space. We believe that moving all of the interrupt handling from the user space to kernel space and eliminating the context switch associated to the execution of an interrupt routine will significantly reduce the response time.

Furthermore, Tock’s design allows us to implement such an approach, as all drivers and capsules are required to obey the split-phase operation principle [53]. This states that all I/O operations in the Tock kernel should be asynchronous and non-blocking. Therefore, there is little chance that a driver or capsule would execute for a long period.

The most straightforward solution is to create a capsule that includes all of the process logic, without any corresponding application. However, this approach has two important disadvantages:

- Building an application as a driver contradicts Tock’s isolation principle, where applications should be deployed separately for safety reasons. Furthermore, this approach brings an overhead in the development process as changes in a capsule translate to recompiling the entire kernel.
- As the kernel is never pre-empted, a capsule that has non-deterministic blocks of code risks blocking the kernel and leading to process starvation.

While implementing a specific capsule for each use-case is not appropriate for our end goal, the solution that we found most suitable was to inject the interrupt routines as bytecode at the kernel layer. However, the main difficulty is preserving the system’s security and having full control over the injected bytecode. Therefore, the requirement is to find a standard bytecode language designed to run in a sandboxed environment. This way, the security of the Tock operating system is preserved as the injected bytecode is proved to be free of any illegal operations and no malicious instructions can be run.

Furthermore, we need to find a bytecode standard where the necessary compilers and execution environments are available and the executor can be integrated in the Tock kernel. As a result, we focused on two main possible solutions: WebAssembly [54,55] and the extended Berkeley Packet Filter (eBPF) [11]. Both are generic assembly languages. However, WebAssembly outlined several issues that made us default to using eBPF.

In the case of WebAssembly, we could not find an executor small enough in size to be integrated in the Tock kernel and still be deployed on low-memory microcontrollers, as the Tock definition states. The only feasible solution was to use wasmi, a module designed to support no-std integration and run on embedded devices [56]. However, due to a cargo issue, compiling wasm with the std dependency disabled was not possible.

Another standard bytecode is eBPF. The extended Berkeley Packet Filter was designed for high-speed processing, first for network traffic and now for any real-time operations inside the Linux kernel. Similarly to WebAssembly, eBPF runs in a sandboxed environment and is a standard bytecode. Considering the disadvatages of WebAssembly and the high-speed processing characteristic of eBPF, the approach that we focused on in this paper is based on eBPF, rather than WebAssembly.

### 3.3. The Extended Berkeley Packet Filter

General purpose operating systems also have certain tasks for which low-latency processing is important. Such an example is the network stack, where, for specific applications, a large number of packets are filtered very quickly. In the Linux kernel, this necessity is solved with the help of the extended Berkeley Packet Filter (eBPF) technology [57].

**Figure 2:**
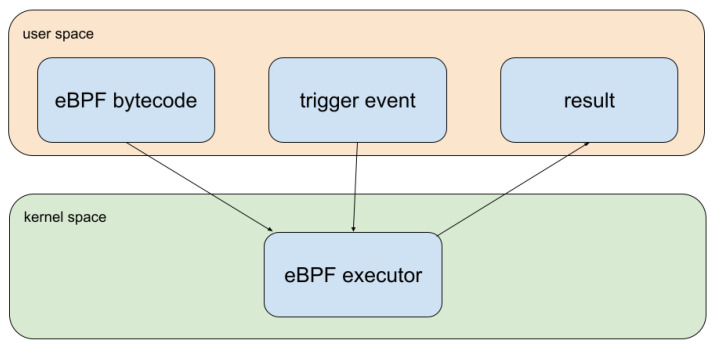
eBPF data flow.

The Berkeley Packet Filter (BPF) is a kernel sandbox capable of running tasks for filtering network data. In 2013, BPF suffered important changes and was renamed eBPF, and can be now used for more varied applications, rather than only network filtering. The main capability of eBPF is to inject specific code into the Linux kernel at the runtime [58], as shown in Figure 2. It is a straightforward way of dynamically introducing kernel code from the user space without recompiling the kernel. eBPF is primarily used for monitoring tasks such as network filtering, security application profiling, and the aggregation of custom metrics [11]. For instance, Netflix uses this technology to optimize and balance network traffic [59].

The bytecode injected in the eBPF sandbox can be obtained from eBPF-specific assembly instructions compiled using bpf_asm. Because of its wide usage, several compilers, such as clang and gcc for C [60] or pyebpf [61] for Python, support compilation to eBPF code. Even Rust can be compiled to eBPF through rustc and LLVM.

The eBPF bytecode is loaded in relation to a specific trigger event and each occurrence of that event triggers its execution. Some of the main advantages of the implementation of eBPF are:

- Fast event processing—the eBPF bytecode is tied to a specific trigger. Therefore, events can be processed with reduced latency, as the whole process takes place at the kernel layer.
- Bytecode verification—as the bytecode is compiled and run in the kernel, the eBPF compiler makes several verifications to ensure that the injected code cannot harm the system. Some of the checks are related to the program not crashing and finishing execution. Non-deterministic blocks of code will not pass this verification.
- Executors—the fact that eBPF is designed to run in sandboxed environments preserves the security of any system that integrates this technology. For instance, executors ensure that the eBPF bytecode does not run forever with the help of an instruction counter.

### 3.4. Integrate eBPF in Tock

The goal of this paper is to propose a secure component for Tock that can ensure low-latency real-time operations. Considering the characteristics of Tock and the safety enforcement resulting from its architecture and implementation, our approach focuses on adding low-latency real-time support to this already secure operating system. The main direction in achieving this is to remove the delays associated with the context switch between the user space and kernel space while also preserving the system’s safety and integrity. The technology that fits these requirements is eBPF, a sandbox designed for the efficient execution of arbitrary code in the kernel.

To preserve the security of the system, we integrated eBPF without the JIT feature. This implies that all injected bytecode is interpreted by the eBPF virtual machine and that any malicious code injected can do no harm to the system. The executor will catch any illegal instruction so the unsecure code is not run, while the kernel and the rest of the applications are not affected. Furthermore, the executor uses an instruction counter that will stop any bytecode execution if a certain limit is surpassed. This ensures that the injected routine cannot block the whole system.

The final goal in using eBPF is to obtain a low-latency real-time system while preserving all of the security characteristics specific to Tock. To this end, we took into account that real-time systems are usually divided into two main categories [62]:

- Hard real-time—the timing constraints, in this case, are very strict and no deadline should be missed. The evaluation of such systems is carried out with a formal approach.
- Soft real-time—these are more tolerant systems, where a certain percentage of missed deadlines is acceptable. These systems can be evaluated experimentally.

Our approach addressed the latter category, and we used an experimental approach to evaluate the results.

## 4. The Implementation

The implementation of the approach presented in this paper focuses on changing the way interrupts are handled in Tock so that the user-space-provided code is executed within the kernel bottom-half interrupt routines. To this end, we implemented the following methodology:

1. Analyze and evaluate the efficiency of the system calls implementation in Tock.
2. Analyze the eBPF mechanism and how it fits into the Tock kernel stack.
3. Integrate the eBPF sandbox and expose an API to user space.

### 4.1. Tock System Calls

The interrupt handling by applications has a high latency in Tock due to the communication and switching overhead between the user space and kernel space and the algorithms used by the scheduler. To understand how applications can process interrupt handlers, we need to briefly present Tock’s system call interface. When a user space application needs to communicate with a kernel capsule, it can achieve this through seven system calls: *yield*, *subscribe*, *command*, *read-write allow*, *read-only allow*, *memop*, and *exit*. Out of these seven system calls, *yield* and *command* are of most interest to us.

*Yield* is the only blocking system call, its only purpose being to stop a process until a task is available in its task queue.

The *command* system call, the system call that requires an action from a capsule, is asynchronous. This means that, when the *command* syscall is made by the user space, the capsule starts the requested action and immediately returns a success/failure value to the user space. The actual action is performed in the background, while the application and other applications can run freely. Whenever the action has been carried out, a notification (upcall) is placed in the user space task queue. Whenever that process yields, the first notification in the task queue will be sent to the application.

In this context, for a process to make a system call and obtain the result, it needs to implement the following steps:

1. Define a callback function that will be called by the kernel when the driver finishes processing and has the result for the desired system call.
2. Issue the subscribe system call to register the callback function in relation to the upcall.
3. Issue the *command* system call to ask the driver to start processing.
4. Yield to wait for the upcall to be triggered and callback function to be called. One *yield* call is associated with a single upcall. If a process has registered callbacks for multiple upcalls in the task queue, it needs to issue *yield* several times.

Using this system of upcalls, a user space may process the result of an interrupt by using the following algorithm:

1. Subscribe to a driver that sends an upcall as a result of an interrupt being triggered.
2. Perform a *command* system call.
3. Perform a *yield* loop. Every time an upcall is scheduled, process within the interrupt within the callback function, and then *yield* again.

This approach has the following drawbacks:

1. It has a significant overhead due to kernel-to-user space switching.
2. The process task queue is limited to a few tasks (10 by default), so, if interrupts are triggered at a high rate, several upcalls will be dropped.

In this approach, the latency is generated mostly from the time gap between the moment the kernel notices the interrupt and the moment the callback function code in the user space corresponding to the upcall issued by the interrupt handler gets executed. The reason behind this is that the process must wait for the scheduler to select the upcall from the process queue, schedule it, and then wait for the kernel to switch the context from the kernel space to user space.

### 4.2. eBPF Sandbox Integration into Tock

**Figure 3:**
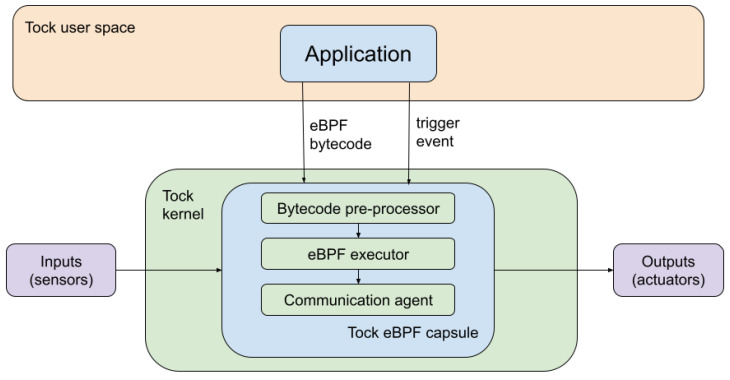
The proposed system architecture.

Considering the bottom-half-only interrupt handling mechanism and the upcall overhead, the implementation that we propose in this paper is to change the existing system calls handling approach and run the code associated with the upcalls using an eBPF executor in the driver. To be more concise, the process will inject the interrupt handling routine in the kernel (driver) instead of registering the callback function for the upcall, as shown in Figure 3.

The injected routine is any eBPF bytecode that the application developer can generate using various tools such as LLVM or gcc. As eBPF is a known standard portable code; this approach ensures that the solution is not dependent on a specific architecture.

The end result is that, once an interrupt is issued, it is handled directly in the kernel space by the driver, without any additional context switches. Since the handling routine is an eBPF bytecode, we can rely on the eBPF sandbox to ensure that it does not block the kernel by running forever. This is necessary as the Tock kernel is non-pre-emptive.

The proposed implementation relies on the following three main components:

- The eBPF executor—this is the capsule that will execute the interrupt handling routine and that needs to interact with the Tock kernel and user space.
- The bytecode pre-processor—modifies the original eBPF bytecode to be compatible with Tock’s memory model.
- The user space communication agent with the eBPF sandbox—this is the capsule’s API through which the defined bytecode is injected into the executor.

#### 4.2.1. The eBPF Executor

The central component in the architecture that we propose is the executor of the eBPF bytecode. This sandbox is integrated into the Tock kernel and runs code injected from the user space. To be more concrete, it is developed as a Tock capsule.

In this context, we identified the following constraints in regard to the executor’s implementation, which are due to Tock’s implementation rules:

- C1—the injected bytecode needs to have a deterministic execution time. It needs to ensure that it finishes executing in a finite amount of time.
- C2—there is no unsafe Rust code allowed at this layer; the executor needs to contain only safe code.
- C3—there is no heap memory allocation allowed in Tock as heap allocation is not deterministic. Therefore, the eBPF executor cannot use the heap.

eBPF is a popular technology and many systems rely on it to create efficient execution contexts. Therefore, there are various open source eBPF executors implemented that are easy to integrate and customize. However, the majority of them are written in C. eBPF executors written in Rust are not that frequent. The research that we made on this topic pointed out rbpf [63] as one of the few open source interpreters written in Rust that are functional and also well-documented.

Rbpf is a small open source project that aims to provide an eBPF executor written entirely in Rust. Besides being well-documented, rbpf supports the complete eBPF instruction set and also has support for features such as just-in-time (JIT) compiling or a disassembler. Although eBPF is originally designed to run kernel code, rbpf is built to run as a user space virtual machine application. This implementation is compatible with all operating systems: Linux, macOS, and Windows.

Generally, eBPF is designed to filter network packets, in which case, the memory operations (e.g., load, store) are made on the packet data. Therefore, the memory area is represented by the area where the packet data are stored. Furthermore, operations can first be made on a different buffer where the packet data are stored—this is the case in the Linux kernel—and then on the actual packet data. Therefore, two pointers are sent to the virtual machine: one to mark the beginning of the memory address and one to mark the end of it.

To support this behaviour, but also other various use cases, rbpf consists of several virtual machines and exposes various customizable features related to memory management, helper functions, or other optimizations. The latest rbpf implementation provides four virtual machines that users can choose from:

- EbpfVmMbuffer—this mimics the running of eBPF bytecode inside a Linux kernel. This is the most common use case. It relies on a metadata buffer, not the actual data buffer, which is passed by the user and is expected to contain pointers to the beginning and the end of the memory area where the relevant data are stored. These data need to be manually copied to an internal buffer.
- EbpfVmFixedMbuff—this is similar to the first virtual machine, with the exception that it handles the metadata buffer rather than manually copying the data. In this implementation, when the execution of the program is started, the internal buffer is automatically populated.
- EbpfVmRaw—this is the most lightweight implementation. It does not simulate a kernel, but simply receives the address of the memory, where the executor uses it directly and no other operations are made.
- EbpfVmNoData—this does not receive any data, and is only for testing purposes.

For our approach, the eBPF sandbox needed to be run in the kernel as a capsule. This led us to outline three main disadvantages in the original rbpf implementation that are not suitable for the use case that we propose. The downsides are presented in relation to the previously defined constraints:

- Rbpf depends on the Rust standard library, which does not exist in the Tock kernel. Furthermore, it relies on the Vec structure, which uses the heap to allocate data. This contradicts C3.
- Rbpf has large blocks of unsafe code. Tock requires that all capsules have zero lines of unsafe Rust code, contradicting C2 from the constraints list.
- The complete rbpf crate is very large in dimensions, as it is designed to run on general-purpose computers. In our case, where we aim to run it on microcontrollers, the available memory is too small to accommodate all the features. Furthermore, many of the nice-to-have features included in rbpf are not necessary for our use case, such as JIT or helper functions. While this is not in direct contradiction with the three constraints defined above, it is an important aspect related to the general purpose of our work.

The advantage of rbpf is that it partially satisfies constraint C1 as the executor carries out some basic correctness verifications. Before running the code, it checks it for incorrect operations (e.g., division by zero), unsupported arguments, incomplete instructions, or infinite loops. However, it does not implement a program flow control verification to ensure that the bytecode is deterministic. As the code is run inside a virtual machine, we can control the virtual machine and force it to stop execution after a certain amount of time, which makes the lack of control flow verification less of an issue, despite being a desirable characteristic.

In this context, we generated a custom version of rbpf, that has the characteristics necessary to be safely integrated into the Tock kernel. To obtain the custom version, we followed some specific implementation steps:

1. Remove all of the unnecessary features, such as helper functions and JIT-related functions. We also trimmed down the virtual machines integrated and kept only one type that is of interest to us. For this first iteration, we used EbpfVmRaw, which does not carry out any kind of pre-processing on memory, but uses it as is. It also does not use any additional buffers: the memory is represented as an array to the bytecode program. This is important from the speed and memory footprint point of view.
2. Rewrite all of the code parts that are dependent on the standard rust library, which appear mainly because the memory is represented as a *vec* structure. To achieve this, we replaced the memory representation with a mutable array of 8-bit unsigned integers.
3. Remove all dereferences of raw pointers, which produce unsafe code that cannot be run inside the Tock capsules.

After the alterations mentioned above, the custom rbpf version has all of the capabilities required to be safely run as a Tock capsule on top of embedded devices that have constrained capabilities.

#### 4.2.2. The Bytecode Pre-Processor

In addition to the eBPF sandbox, an important aspect in running safe custom code in the kernel is how the eBPF bytecode to be executed is generated. For this, we need to take into account the constraints related to Tock and to the hardware capabilities. While, in general, eBPF is designed to run on computers, in our case, we needed to adapt to microcontrollers that have a reduced processing power and memory.

**Figure 4:**
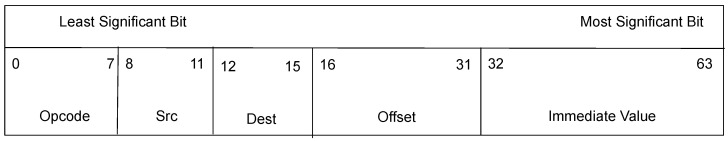
eBPF instruction format.

The eBPF ISA is straightforward, the binary program itself being a long sequence of 64-bit instructions that must respect the format that is presented in Figure 4.

The 64-bit instruction is split into the following fields:

- Eight-bit Operation Code (Opcode)—this specifies the instruction that must be run by the machine (e.g., load, store, ALU operations, etc.). Originally, until Linux version 5.3, eBPF did not support running loops in order to make sure that the program ends (the kernel checks the control flow graph for back edges), but, currently, the jump instructions are allowed.
- Four-bit Source Register (Src)—this is a register where values can be read. This register can be used or not depending on the instruction.
- Four-bit Destination Register (Dest)—this is a register where values can be stored. This register can be used or not depending on the instruction.
- Sixteen-bit Offset—This is used especially by load and store instructions.
- Thirty-two-bit Immediate Value.

The bytecode pre-processor is part of the rbpf sandbox that we used for our implementation.

The major alteration is related to the memory buffer allocation. Originally, the executor represented the memory area passed from the user space as a *Vec* structure, which is allocated on the heap at the runtime. For our use-case where the executor is represented as a Tock capsule, this contradicts C3. Therefore, we replaced the original memory management implementation in order to replace the *Vec* struct with an *array*, which is allocated on the stack.

Another feature of interest related to memory management is that the initial rbpf implementation uses a *Vec* structure for the virtual machine’s stack. As we stated before, this contradicts C3, so we removed it. Our solution was to allocate an *array* in the main capsule and pass it to the executor together with the memory structure. The two were concatenated and passed further on. Therefore, one of the challenges is to ensure that operations related to memory management are carried out correctly.

#### 4.2.3. The User Space Communication Agent with the eBPF Sandbox

**Figure 5:**
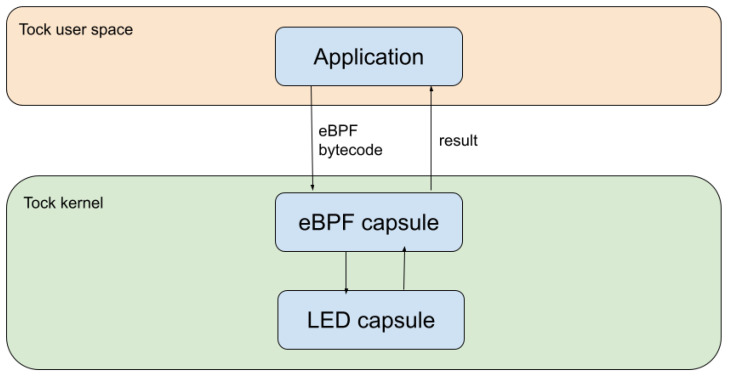
The communication between the application and a peripheral capsule using the eBPF executor.

The final component of the proposed architecture is related to the actual integration of the eBPF executor in the Tock stack. The sandbox was deployed as part of the kernel space, but also communicates with the user space and exposes a user space API (Figure 5, which makes the integration more complex. At the kernel layer, the rbpf executor module was included in a custom Tock capsule that was designed to intermediate this communication.

The capsule that we created is meant to offer a generic implementation that allows for interactions with all other existing Tock capsules. In this context, this capsule does not control the hardware directly, but sends commands to already implemented hardware control capsules. However, it needs to associate the bytecode to the necessary hardware operations, identify the appropriate hardware control capsule, and define the necessary commands. Further on, it reads the result that the hardware control capsule returns and sends it back to the user space.

For the communication with the user space, the capsule receives the bytecode from the application via a command and allows for system calls. The actual bytecode is passed as a byte array stored inside a buffer shared by the kernel and the application. This relies on the Tock standard of sharing data between the user space and the kernel.

## 5. Tests and Results

To evaluate the efficiency of the proposed approach, we measured the system’s latency when handling interrupts. As we target obtaining a soft real-time system, the necessary evaluation approach relies on monitoring the system’s behaviour and measuring latencies.

In order to measure the efficiency of our implementation, we made latency measurements for the initial Tock implementation that involves user-space–kernel-space transitions, and for our proposed approach, where interrupts are handled in the kernel.

To generate the eBPF bytecode for the tests, we analyzed the existing eBPF assembly language that was defined to make the bytecode readable. However, there is no standard instruction set. The assembly language is mostly used by dissemblers to understand the binary code more easily because there is no official assembler program and the usage of inline eBPF assembly in C code may fail for various instructions. The downside is that various assemblers define different instructions, which was a problem for us.

For our use case, we used LLVM to generate eBPF bytecode from C code. However, only 30% of the tests were generated this way. For the rest of them, some of the operations that we used could not be generated using LLVM. As we have no control over the bytecode that LLVM generates, we could not find the correct C code that would generate the bytecode instructions that we aimed to test. Therefore, for the rest of the tests, we chose to write direct eBPF binary code defining the specific needed instructions.

### 5.1. The Evaluation of the Original Implementation

The first tests that we carried out were related to the performance of the Tock operating system on various architectures. These tests were meant to identify the original latencies in the Tock kernel and the overall system behavior in handling external triggers. To achieve this, we implemented two test categories:

1. Overall behaviour—these are stress tests meant to identify if a device running Tock can handle a massive amount of high-frequency triggers.
2. Latency measurement—these tests focused on evaluating the latency in handling interrupts in Tock.

For all of the tests, we used a BBC micro:bit v2 device, which has an nRF52833 MCU. This MCU has a frequency of 64 MHz and is one of the slowest MCUs supported by Tock; however, the micro:bit v2 is one of the most popular devices that is completely supported by Tock.

As the tests involve generating high-frequency triggers, we used an oscilloscope to carry that out.

#### 5.1.1. Overall Behaviour Tests

For these tests, we set the oscilloscope to generate alternative HIGH-LOW values at different frequencies, while the micro:bit ran one or more processes that handle the received interrupts. The interrupt handler routine is defined for both edge triggers and, when called, increments a value and prints it in the serial console.

For the case when the micro:bit runs one application whose only target is to handle these incoming interrupts, we managed to handle all triggers received at a frequency of 2 KHz. For higher frequencies, around 30% of the interrupts are lost. Finally, at a frequency of 10 KHz, the system does not print any message, as it is too fully occupied to handle the interrupts coming from the oscilloscope, and the print function never gets called.

We made the same test with a system that runs two parallel applications in order to simulate a real use-case in which multiple applications run at once. This enabled us to measure the benchmark application’s responsiveness to interrupts while another I/O intensive process is running. Therefore, we ran the previously mentioned application that handles interrupts in parallel with an application that makes an LED blink once per second. The interrupt frequencies at which the system works are the same, while, for frequencies higher than 2 KHz, up to 70% of the interrupts are lost. Similarly to the first case, for interrupts generated at a frequency of 10 KHz, the system stops printing messages; however, the LED blink process still functions.

The final test replaces the LED blink application with one that registers an interrupt routine for a button. In this case, for interrupts generated at a frequency of 2 KHz, the overall behaviour apparently shows that both routines, the one for the pin connected to the oscilloscope and the one for the pin connected to the button, are called. We still need to investigate the actual behaviour that leads to this appearance.

The conclusions of these tests are that Tock is not designed to handle high-frequency interrupts and that the interrupt handler mechanism is not optimized for fast responses.

#### 5.1.2. Latency Measurements

To evaluate the interrupt handling latencies specific to Tock, we clocked the response time between syscalls. To implement the tests, we created a kernel capsule that acts as the system monitor. It implements a timer and receives all system calls to be monitored.

All of the measurements were computed as an average of 150 different samples. The variance in the measurements was around 150 µs.

At the user space layer, we implemented four scenarios:

1. One user space process—the device runs one process that continuously issues a command syscall every 250 ms.
2. Three identical user space processes—the device runs three different processes, each issuing a command syscall every 250 ms.
3. One CPU-intensive process—to put more pressure on the system, it runs one CPU-intensive process (a loop without any delays) and two processes that issue a command syscall every 250 ms.
4. Two CPU-intensive processes—this stress test uses two CPU-intensive processes and one blocking process, similar to the ones presented above.

For each scenario, we performed two different measurements: one for a synchronous syscall and one for an asynchronous syscall.

The synchronous measurement focuses on the time it takes for a syscall to be sent from the user space to the kernel and for the user space to receive the result. To measure this, we implemented a user space function that calls a command twice, consecutively. The capsule starts a counter right before answering the first command and stops it when the second command reaches the kernel. Therefore, we are computing the kernel—user space—kernel trip.

The asynchronous measurement clocks the time it takes for a syscall to reach the kernel and get back to user space, but in the case of an asynchronous capsule. To implement this, we created a user space function that first calls subscribe for a capsule result, and then calls command. In the capsule, the timer is started once the command reaches the kernel, and, immediately after this, the capsule calls the user space function that is subscribed to it using an upcall. Once this is called, another command is issued. When this syscall reaches the kernel, the timer is stopped.

The measurement results are displayed in Table 1. The delays obtained are considerably higher than the delays specific to a low-latency real-time system, where the values are around 50 μs [64].

### 5.2. The Evaluation of the Proposed Approach

The evaluation of the system implemented was carried out incrementally. Specific components of the system were benchmarked, as well as the overall solution. In this context, we can divide the tests into two categories:

- Correctness tests—necessary for evaluating the correctness of the implementation;
- Performance tests—used to evaluate the efficiency of the proposed solution.

#### 5.2.1. Correctness Evaluation

A major implementation effort is related to the development of the eBPF executor compatible with the Tock kernel. This is based on the rbpf [63] Rust crate, but significant changes were made to the initial version of the module, especially related to memory management. Therefore, we created a testing framework to ensure that all if the operations that involve memory management in eBPF (load and store), where major changes were made, are correctly implemented.

#### Custom Test Framework Implementation

The first tests focus on comparing results when running eBPF bytecode using the original rbpf implementation to the one we adapted. This is necessary to ensure the correctness of the solution we propose.

To this end, we created multiple C applications (listed below) for each load and store operation, compiled them to eBPF, then ran the bytecode in a Rust application [65]. This allowed us to compare the rbpf output to the output obtained from our modified rbpf implementation. These tests were run on a general-purpose system, capable of running both versions of the rbpf implementation.

**#include** <stdio .h>
**#define** SEC(NAME) _ _attribute_ _((section (NAME), used))
 
*//LD_ST_DW_REG*
**struct** _ _sk_buff {
   **long long** len;
   **int** mark;
   **int** ifindex;
   **int** queue_mapping;
};
 
SEC( "segment ,. classifier" )
**unsigned long long** sample_func(**struct** _ _sk_buff ∗skb) {
   **unsigned long long** first = 2 ∗ skb->len + 2;
   skb->len = first;
   **return** skb->len;
}
 
*//LD_ABS_DW*
**struct** _ _sk_buff {
   **unsigned long long** len;
   **int** mark;
   **int** ifindex;
   **int** queue_mapping;
};
 
SEC("segment,. classifier")
**unsigned long long** sample_func(struct _ _sk_buff ∗skb) {
   *// asm volatile ("r0 = ∗(u8 ∗)(%0 + 2)":"=r"(sub + 5));*
   **long long** x = (**long long**) ∗((**long long**∗)(skb + 1));
   asm( "r0␣=␣∗(u32␣∗) skb [2]" );
   **return** x;
}

For the rest of the four tests, LLVM did not generate the eBPF bytecode for the required instructions. Therefore, we manually generated the binary bytecode based on the ALU instructions [66]. For instance, for the test ST_DW_IMM, where we store a double-word value from an absolute indexed address, the injected bytecode is *72 10 03 00 ff 00 00 00*, where *0x72* represents the instruction stb[dst+off], imm *(uint8_t *) (dst + off) = imm, *10* is the actual *10* value, and *03* is the *r3* register.

#### eBPF Executor Efficiency Tests

The testing framework for the correctness of the implementation was also used to evaluate the efficiency of the eBPF executor that we propose. We used the same framework to measure how fast the load and store instructions run in the original rbpf executor compared to the one that we propose.

Table 2 outlines the results, where the timings obtained by the eBPF executor that we implemented are, on average, 3 to 4 times lower for simple operations and around 2.5 times lower for more complex ones. These results are because the rbpf implementation relies on the std-defined type *vec*, which executes many additional function calls for each operation. Since the executor that we implemented removed all blocks of code related to the standard library, the execution is faster.

#### 5.2.2. Complete Platform Tests

For the evaluation of the complete proposed approach, we used the same micro:bit v2 device as in the initial tests. We defined several sets of tests to evaluate both the system’s response to interrupt hammering and the delay in interrupt handling. For all of the tests, we used an oscilloscope to generate an oscillating signal. For each test, we performed 50 measurements and computed the average value, which is presented below. The variance in the obtained results was 5 µs.

First of all, we tested the system’s responsiveness when receiving interrupts at a frequency of 10 KHz. In the initial tests, without the eBPF executor integrated, at this frequency, the system stops printing debug information. During these current tests, all interrupts coming at a frequency of 10 KHz were successfully handled.

**Figure 6:**
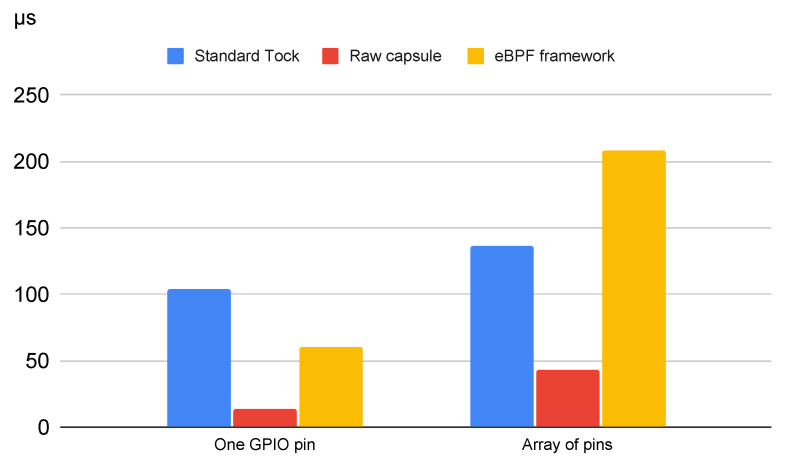
Interrupt handling delays.

Further on, we focused on two main test categories to evaluate the response time. These tests were conducted to compare the response time of the eBPF-based approach with the response time of the standard Tock approach and with a capsule designed to handle the same interrupts. The results are outlined in Table 3 and in Figure 6.

First, we implemented the standard approach, which involves writing an application that registers a callback for when an interrupt comes on a GPIO pin and modifies another pin.

For the second test, we created a simple capsule that reads interrupts coming on a statically defined GPIO pin, reads one value returned by the eBPF executor once it finishes processing the bytecode, and writes it on a predefined GPIO pin. The bytecode injected from the user space reads a value and returns the same value. To summarize, when one of the pins is set to HIGH using the oscilloscope, the other pin is set to HIGH and vice versa. In this case, the time span from when the signal is set to high to when the new value is written on the GPIO pin is 60 µs. As a comparison, when implementing the same logic using a simple capsule, without the eBPF execution overhead, the response is 14 µs.

The final set of tests targets a more complex approach. This use case relies on implementing the same behaviour as the first set of tests, but it works with all available device pins, not only two predefined ones. In this case, the capsule passes an array structure where each pin has attributed an index equal to the pin number. When an interrupt comes on any of those pins, the bytecode is executed. Similarly to the first use case, the eBPF bytecode receives a vector and returns the same vector with one value changed. The main difference is that the capsule has to handle an array, not a simple value, and it has to iterate the array to identify the pins altered by the eBPF bytecode. Further on, it writes the new values on the corresponding pins by calling the GPIO control capsule specific to the device.

In this case, the response time is 208 µs. The eBPF code execution takes 43 µs, whereas the rest of the time is spent by the capsule handling the array.

The results obtained by the approach that we suggest were compared to the delays measured in the original Tock implementation and to the ones resulted in the case of a dedicated capsule to handle these interrupts. As expected, the delays in the case of a dedicated capsule are the lowest, as all of the application logic is implemented in the kernel.

When compared to the original Tock implementation, the eBPF-approach is faster for the first use case, which is when the GPIO pins are predefined. This is because the integration of the eBPF module reduces the context switch overhead. However, in the second use case, when an array of pins is used, that response time increases significantly. This is due to the operation of iterating the array. In Rust, iterating an array is very time consuming. Therefore, the main future improvement that we will focus this research on is to reduce this overhead.

### 5.3. Results

The final results outline a significant improvement in the system’s response to high-frequency interrupts. To be more precise, we implemented an approach that allows the Tock kernel to run custom code triggered by interrupts coming at a frequency of 10 KHz, while the original kernel freezes during such a use case.

Initially, the system could not handle interrupts coming at a frequency higher than 2 KHz and, for interrupt frequencies as high as 10 KHz, the console would not print anything. In comparison, when the interrupts were handled using the eBPF executor that we propose in this paper, all interrupts were successfully handled, even the ones coming at a frequency of 10 KHz.

When compared to the raw approach of introducing a custom capsule in the kernel meant to handle specific interrupts, the eBPF-based solution has a lower response time. However, the main advantage of the proposed solution is its generality. It does not involve a different capsule for each different use case but allows the interrupt routine to be injected in the kernel from the user space.

Regarding the delay in handling an incoming event, the approach that we propose measures a mean value of 200 µs between the interrupt being triggered and a pin’s status being changed. This value is for the case where we work with an array of pins that are being read. If we resume to a specific use case where the pins are statically defined in the capsule, the delays drop to 60 µs, which is comparable to other real-time systems. The evaluation made by Zhang M. et al. [64] outlines that a real-time system implemented using a Raspberry Pi 3 with a 1.2 GHz CPU and a BeagleBone Black with a 1 GHz CPU has a response latency between 45 and 75 µs.

## 6. Conclusions

The purpose of this paper is to present a novel approach toward a secure low-latency operating system for constrained devices. Currently, systems that need to ensure soft real-time responses rely on real-time operating systems that are proven to be less secure from a memory management point of view. One of the safety perils that existing RTOSs such as FreeRTOS or Zephyr have is due to the fact that they are written in C, where writing C code can easily lead to memory management vulnerabilities.

In this context, we analyzed the state-of-the-art operating systems for embedded devices that are built using another language. One of the most mature and used operating systems is Tock, whose principal characteristic is that it is written in Rust and clearly separates the kernel from the user space, which ensures a high degree of safety. The only downside is that Tock is not designed to handle interrupts with a low latency.

The approach that we proposed is to optimize Tock so that it can handle soft real-time interrupts and achieve a secure embedded operating system that can handle events with a low latency. To this end, we integrated eBPF in the Tock kernel and leveraged a technology that is mainly used for network traffic filtering and kernel monitoring to execute interrupt handlers with a fast response.

When compared to the measurements in delay that the original Tock kernel has, our approach was three times faster.

So far, our work is at a prototype stage and has validated that executing the interrupt handling routines using an eBPF sandbox lowers the response time of the system and makes it more stable to interrupt hammering.

Further on, our work will focus on reducing the overhead specific to array iteration. This is the cause for the large delay in handling an array of pins and this is what we aim to make more efficient. Once this issue is handled, we will focus on building a full framework that integrates various eBPF bytecode generators so that developers can define the interrupt routines without the need for interacting with third-party tools. The framework will include tools such as LLVM and gcc to generate the bytecode instead of the programmer. Furthermore, we aim to integrate JIT support to increase efficiency. However, this requires additional complex verifications in place to preserve the system’s safety.

Finally, our aim is to provide this platform as an open source addition to the Tock kernel that the community can benefit from.

## Figures and Tables

**Table 1:** Latency measurements using a micro:bit device.

	One User Space Process	Three User Space Processes	One CPU-Intensive Process	Two CPU-Intensive Processes
Synchronous measurement	5127 µs	90,127 µs	91,452 µs	125,250 µs
Asynchronous measurement	9213 µs	91,545 µs	90,643 µs	120,903 µs

**Table 2:** Load and store operations execution speed comparison between the rbpf executor and the proposed executor.

Test Name	Description	Rbpf Executor	Proposed rpbf-Based Executor
LD_ST_DW_REG	Load and Store Double-Word into Reg	2701 µs	660 µs
LD_ABS_DW	Load Double-Word from absolute indexed address	1415 µs	490 µs
ST_DW_IMM	Store Double-Word to absolute indexed address	1986 µs	500 µs
LD_IND_DW	Load Double-Word from indirect indexed address	1698 µs	293 µs
Stack test	Generate a vector of 496 char elements on the stack with values from 0 to 495	75,159 µs	28,358 µs

**Table 3:** Comparison between delays obtained in handling interrupts.

	Standard Tock Approach	Raw Capsule	eBPF Framework
One GPIO pin	104 µs	14 µs	60 µs
Array of pins	136 µs	43 µs	208 µs

## Abbreviations

The following abbreviations are used in this manuscript:

ALU	Arithmetic Logic Unit
eBPF	Extended Berkeley Packet Filter
GPIO	General Purpose Input Output
IPC	Inter-Process Communication
MCB	Microcontroller Board
MCU	Microcontroller Unit
MPU	Memory Protection Unit
OS	Operating System
RTOS	Real-Time Operating System
SBC	Single-Board Computer
